# Virtual reality video game improves high-fidelity memory in older adults

**DOI:** 10.1038/s41598-021-82109-3

**Published:** 2021-01-28

**Authors:** Peter E. Wais, Melissa Arioli, Roger Anguera-Singla, Adam Gazzaley

**Affiliations:** 1 Department of Neurology, Neuroscape and Weill Institute for Neurosciences, University of California, San Francisco, UCSF-MC0444, 675 Nelson Rising Lane, San Francisco, CA 94158 USA; 2 Departments of Physiology and Psychiatry, University of California, San Francisco, San Francisco, USA

**Keywords:** Psychology, Neuroscience, Cognitive ageing, Cognitive neuroscience, Learning and memory

## Abstract

Therapeutic interventions have not yet been shown to demonstrate restorative effects for declining long-term memory (LTM) that affects many healthy older adults. We developed a virtual reality (VR) spatial wayfinding game (Labyrinth-VR) as a cognitive intervention with the hypothesis that it could improve detailed, high-fidelity LTM capability. Spatial navigation tasks have been used as a means to achieve environmental enrichment via exposure to and learning about novel and complex information. Engagement has been shown to enhance learning and has been linked to the vitality of the LTM system in the brain. In the current study, 48 older adults (mean age 68.7 ± 6.4 years) with average cognitive abilities for their age were randomly assigned to 12 h of computer game play over four weeks in either the Labyrinth-VR or placebo control game arms. Promptly before and after each participant’s treatment regimen, high-fidelity LTM outcome measures were tested to assess mnemonic discrimination and other memory measures. The results showed a post-treatment gain in high-fidelity LTM capability for the Labyrinth-VR arm, relative to placebo, which reached the levels attained by younger adults in another experiment. This novel finding demonstrates generalization of benefits from the VR wayfinding game to important, and untrained, LTM capabilities. These cognitive results are discussed in the light of relevant research for hippocampal-dependent memory functions.

## Introduction

Declining long-term memory (LTM) impacts diverse aspects of cognitive performance and overall quality of life for many healthy older adults^1–4^. Chronic memory loss is typically first apparent as reduced capability for high-fidelity memory^5^, which is the most precise form of LTM. High-fidelity memory depends upon flexible association of diverse bits of information for facts and events that are remembered in distinct and detailed terms^6^, and it can be conceptualized in terms of source^3,5,7^, associative^8,9^ and autobiographical memory^10,11^, as well as mnemonic discrimination as a behavioral task to operationalize pattern separation processes^12,13^. To our knowledge, there has yet to be any cognitive or pharmaceutical interventions that have demonstrated restorative effects for the decline in high-fidelity LTM that occurs with aging.

Encoding and retrieval of high-fidelity LTM depend upon hippocampal processes^14^, which are often disrupted in normal aging^15^. These hippocampal memory processes have been examined across the human lifespan with adaptations of a behavioral pattern separation task^16–18^. The results from a growing literature show a decline in mnemonic discrimination capability for nondemented older adults^1^, as well as associated aging-related changes in functional activity in the hippocampal region^19^.

The potential to stimulate plasticity and up-regulate function in the hippocampus has been demonstrated in rodent model studies using spatial exploration of novel, complex environs^20–23^. Specifically, spatial navigation tasks have been applied to achieve environmental enrichment in animals^24–27^, which refers to exposure and learning of novel and complex information that have been linked to the vitality of the memory system in the brain^21^. Moreover, in a study with younger adults, who played five-hours in a visually complex video game, participants improved their capability for high-fidelity LTM, relative to placebo control^28^.

**Figure 1:**
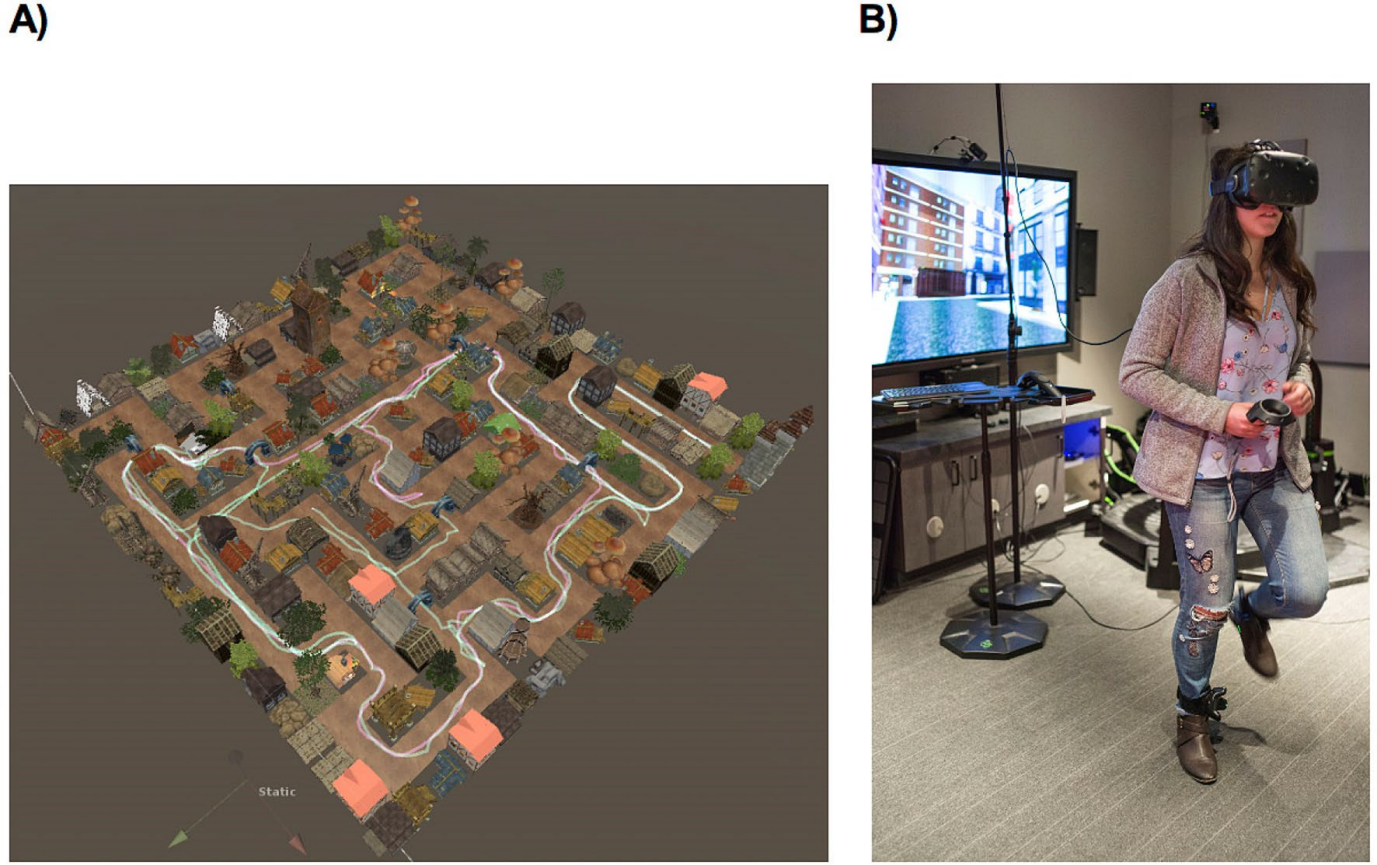
Illustrations of the virtual reality game in Experiment 2 (A) Via a head-mounted virtual reality display, participants had a first-person view of wayfinding trials. The game goal was to learn and efficiently complete errands, as illustrated here with the overhead perspective of the feedback screen from a Village neighborhood. (B) Game movement was effected by a participant’s ambulation, which was co-registered into the game map from ankle-mounted tracking sensors.

We developed a virtual reality (VR) spatial wayfinding game as a cognitive intervention aimed to mimic environmental enrichment approaches used in the study of animals with the goal of improving, and potentially restoring, memory abilities in healthy older adults. Using a head-mounted VR display (i.e., HMD VR), the Labyrinth game immerses participants in entirely novel, visually elaborate environments (Fig. 1A). Applying video games as cognitive interventions to impact behavior and brain function is an emerging method in translational neuroscience research^29^, and early results suggest mixed findings. Successful interventions have depended upon how effectively this demanding technology was targeted to engage and sustain the attention of participants during their tasks^30,31^. Importantly, using an HMD VR drives greater engagement and increased subsequent success for participants, relative to their performance on the same task using a flat-screen-monitor version^32–34^.

In the Labyrinth game, the three-dimensional (3D) functionality of VR enabled synchronization of participants’ head movements (i.e., direction of gaze) and associated walking motion to effect game movement. The game presents engaging, adaptively increasing challenges to learn virtual urban and village neighborhoods and then navigate through them to complete assigned errands. Advancement through the treatment regimen requires participants to acquire efficient, allocentric navigation capabilities so they can demonstrate above-threshold wayfinding performance in the just-learned, novel neighborhoods. Here, we report results for a cohort of healthy, cognitively average older adults, who were randomly assigned to either active training with Labyrinth-VR on a head-mounted display, or placebo control games on a handheld computer with a set of narrative adventure games.

Another novel element in our intervention to improve high-fidelity LTM capability incorporates participant ambulation as the means to effect movement in the game (i.e., walking in place as illustrated in Fig. 1B). Two advantages of game movement via ambulation are increasing the immersive qualities of the wayfinding experience via a more ecologically valid engagement method and evoking the peripheral nervous systems during the treatment. Mild, acute exercise has also been linked with benefits to cognitive performance generally, as well as to enhanced hippocampal function^35–37^. These beneficial effects on LTM performance and cognitive health have been attributed to evidence that physical exercise increases cerebral blood flow (CBF) and up-regulates hippocampal plasticity^35,37–39^.

An emerging consensus in the memory literature finds that encoding and retrieving different types of high-fidelity memories depend upon multiple hippocampal subregions and their linked functional networks with cortex^6,40–44^. Importantly, episodic, spatial, associative and temporal order are all types of high-fidelity LTM memories that are required for efficient spatial wayfinding. Convergent with this framework for hippocampal function in spatial navigation and high-fidelity LTM^45^, humans with unilateral hippocampal damage demonstrated impaired capabilities in spatial planning and allocentric navigation^46^.

Our motivation was to develop and demonstrate a cognitive intervention based on spatial wayfinding through unfamiliar surroundings that induced beneficial gains in untrained measures of high-fidelity LTM, which were assessed promptly before and after treatment. The wayfinding game engaged participants in an immersive and interactive experience through HMD VR technology^31^, which led them through approximately 15 h of an adaptive challenge to learn and remember details in novel, virtual environments.

In order to establish baseline results on a primary measure of high-fidelity LTM, Experiment 1 compared high-fidelity LTM performance between younger and healthy older adults using a mnemonic discrimination task (i.e., MDT)^1^. We adapted MDT for our research from a well-established test of behavioral pattern separation task that is thought to depend on hippocampal functions^6,14,15^ . Then with a separate group of healthy older adults, Experiment 2 examined the treatment-induced effects of Labyrinth-VR on this primary memory outcome measure, MDT, as well as two other memory measures (i.e., recent autobiographical memory and spatial recognition). We expected the comparison of MDT results between Experiments 1 and 2 to show: (i) replication of baseline performance for healthy older adults between two groups, and, importantly (ii), whether hypothesized training-induced gains in high-fidelity LTM for healthy older adults would restore their performance to a level on par with younger adults.

## Experiment 1. MDT with older and younger adults

### Methods

#### Participants

Twenty-seven healthy younger adults between the ages of 20 and 29 years (13 males) who were native speakers of English, screened to be free from any psychotropic or thyroid medications, and had completed 14 or more years of education, gave their informed consent in accordance with the Institutional Review Board of the University of California, San Francisco (UCSF), and received a $20/hour fee in compensation for approximately 2 h of their time needed to complete the study procedure. All methods were carried out in accordance with relevant guidelines and regulations for experimental protocols approved under UCSF IRB #15-16824. All participants had normal or corrected-to-normal vision. Two younger participants’ data were excluded from analysis: in one case because recognition memory was at the level of chance, and in the other case because the participant fell asleep during one block of the task.

Twenty-six healthy older adults (12 males, mean age = 70.5 ± 5.6 years), who had completed a minimum of 12 years of education and otherwise met the same criteria as the younger adults, gave informed consent and received the same fee. One older participant’s data were excluded from analysis because of failure to comply with test instructions.

Because participants in Experiment 1 were enrolled on the basis of their age, and not assigned between the younger and older groups at random, the experiment is a quasi-experimental design.

#### Neuropsychological testing

All older adults enrolled in the study were, at the time of recruitment, administered 15 standardized neuropsychological tests that assessed executive and memory functions, and depression. Inclusion criteria for each participant were that all their tests scored ≥ -1.5 standard deviations of normative values for their age. These evaluations were conducted by UCSF Neuroscape within six months of study recruitment and included the following tests: MMSE^47^, geriatric depression (GDS)^48^, working memory and verbal learning (CVLT-II)^49^, processing speed (WAIS-R)^50^ , visual-motor sequencing (DKEFS Trail-Making A and B), executive function (DKEFS Stroop interference test), semantic fluency and phonemic fluency (DKEFS)^51^. Table 1 summarizes the older participants’ mean scores in the standardized cognitive assessments relevant to LTM, which were collected in the neuropsychological evaluation battery.

**Table 1:** Standardized cognitive assessments.

	Experiment 1	Experiment 2	
	Older adults	Labyrinth	Controls
**CVLT word list**			
Total recall	10.88 ±2.47	10.84 ±2.62	12.23 ±2.00
Total intrusions	1.02 ±1.40	0.80 ±1.36	0.45 ±0.80
**CVLT long delay**			
Free recall	11.17 ±2.24	9.71 ±2.99	11.73 ±2.51
Intrusions	0.52 ±1.06	0.57 ±1.08	0.45 ±0.74
Cued recall	11.67 ±2.12	10.71 ±2.31	12.05 ±2.21
Intrusions	1.18 ±1.20	0.81 ±1.36	1 ±1.15
Digit symbol seconds	66.33 ±10.50	63.76 ±18.69	62.32 ±12.88
Number/letter seconds	85.77 ±38.49	89.67 ±54.07	81.51 ±34.71
Errors	0.45 ±0.93	0.14 ±0.36	0.68 ±1.25

Summary of participants’ mean scores in a selection of standardized cognitive assessments relevant to LTM, which were collected in a neuropsychological evaluation battery conducted within six months of study recruitment. All participants performed all tests in the neuropsychological battery at or above − 1.5 SD of age-normalized levels. These data are presented separately for the Older Adults in Experiment 1, and the Labyrinth and Controls arms in Experiment 2. There were no differences between the three groups in mean performance on any of the tested cognitive measures.

#### Discrimination task stimuli

211 images of common objects were presented, in color and centered on a white screen. The images were displayed at 768 × 1024 pixel resolution for viewing on a 15-inch diagonal LCD from a distance of approximately 23 inches. 180 stimulus pairs were organized as 90 target objects associated with 90 very similar lures, and 18 images were distinctly novel objects. The remaining 13 stimuli were presented to orient participants to the separate encoding and test procedures (i.e., five targets, five paired lures and three novels). The procedures were presented using PsychoPy version 1.82.01 (copyright J.W. Pierce, 2002–2015).

#### Procedure

An incidental encoding task was presented as a study phase, and then after an interval of 30 min, an old/new recognition task was used in a test phase (Supplementary Fig. 1). These stimuli and experimental procedures have also been employed in two of our other published original research articles^6,44^.

In the study phase, participants viewed one target image on each trial (stimulus presentation = 2.5 s) while responding to incidental questions designed to promote in-depth visualization of each object: (1) “yes or no, will the object fit inside a lady’s medium shoe box?”; and (2) “yes or no, can you carry the object across the room using only your right hand?”. The two questions were presented in separate runs so that participants viewed each target image twice. Participants were kept naïve to the memory test until being instructed during the practice procedure before their first test block.

Each of three test blocks included 66 trials and was divided into two segments such that participants were cued at the mid-point by on-screen instructions to take a brief break (7 s). 30 targets, 30 lures and six novel items were presented in each block, and the total run time of each block was 5 m 37 s. Presentation of target, lure and novel trials was randomized within each block, and block order was counter-balanced across participants. A target and its paired similar lure were never presented in the same test block, and the order of target first or similar lure first was counter-balanced across all the trials.

The trial procedure presented one object image (2 s), which was followed immediately by a response screen (2 s) that cued the participant to enter an old/new recognition rating on a response box held in the right hand. Participants were instructed to respond whether each item was 1 = definitely old, 2 = maybe old, 3 = maybe new, or 4 = definitely new. Fixation (1 s) separated each trial. Considering the 30-min break between study and test phases, and the average duration of the test phase, the approximate retention interval was 50 min running from the mid-point of the study phase to the mid-point of the test phase.

For analysis, trials were later sorted on the basis of participants’ responses into hits (“old” responses to targets), misses (“new” responses to targets), lure correct rejections (LureCR: “new” responses to lures), lure false alarms (LureFA: “old” responses to lures), novel correct rejections (NovelCR: “new” responses to novel items), and novel false alarms (NovelFA: “old” responses to novel items). Analysis of the distribution of confidence ratings associated with old/new responses was not a focus in this experiment and is not reported here.

The analysis of high-fidelity LTM performance used each participant’s LDI, which indicated their discrimination of the lures as new during the MDT. Recognition of a target as “old” could be based on underlying processes for discrimination, for memory of the gist of the object, or, most likely, contributions from both processes^6,44^. Correct rejection of a lure as “new,” however, indicates a memory judgment based on underlying discrimination (i.e., any given correct rejection may have identified differences between a lure and its paired target, or between a lure and all other objects presented in the procedure).

By augmenting the simple proportion for responses to lures based on discrimination (i.e., Lure CR) with subtraction of the proportion of false alarms to an independent class of novel stimuli (i.e., Novel FA), the LDI provides a more specific scale for each participant’s effectiveness in discriminating details in memory and for how their effectiveness may have been influenced by their overall response bias in the old/new task (i.e., bias to respond “old” to targets and to lures^52^.

### Results

The accuracy of responses for targets, lures and novels were compared by stimulus category between the groups of younger and older participants (Table 2). From these results, a mean Lure Discrimination Index (LDI) was calculated for each of the groups, such that LDI = (proportion Lure CR—proportion Novel FA).

**Table 2:** Accuracy and Discrimination in Experiment 1.

	Targets	Lures	Novels	LDI
	Hit	CR	CR	
Younger	0.81 ± 0.02	**0.68 **± 0.02	0.99 ± 0.01	**0.67 **± 0.02
Older	0.80 ± 0.02	**0.55 **± 0.03	0.98 ± 0.01	**0.53 **± 0.03

Summary statistics show the accuracy of responses for targets, lures and novels in the MDT for groups of younger and older adults in Experiment 1, as well as the calculated LDI for each group (i.e., proportion Lure correct rejections—proportion novel false alarms). CR indicates correct rejection. Values in bold face indicate differences between groups in that measure, *p* < 0.001.

The critical comparison of capability for lure discrimination between groups found that mean LDI for younger adults was superior to that for older adults (*t*_48_ = 3.65, *p* = 0.001, Cohen’s *d* = 1.12, between groups test with unequal variances). The lower LDI for our cohort of healthy older adults is evidence of their diminished capability, on average, for mnemonic discrimination— a key element in high-fidelity LTM retrieval.

## Experiment 2. Labyrinth-VR treatment with older adults

### Methods

#### Participants

49 older adults (mean age 68.7 ± 6.4 years, 20 females) with average cognitive capabilities for their age participated in this intervention study. Inclusion criteria were: native speakers of English, completion of 12 or more years of education, normal or corrected-to-normal vision, physical stamina sufficient to comfortably walk 40 min at a brisk pace on level ground, dexterity sufficient to comfortably operate a computer game hand controller, freedom from any psychotropic medications and/or conditions contra-indicated for fMRI, no history of vestibular system problems, vertigo or frequent dizziness, and limited if any experience with regular computer game play. In order to comfortably fit within the railing of the ambulation platform, physical criteria were height between 60″ and 73″, inclusive, and maximum waist size of 42″. Participants gave their informed consent in accordance with the Institutional Review Board of the University of California, San Francisco, and received a $20/h fee in compensation for approximately 22 h of their time expended to complete all the required study procedures. All methods were carried out in accordance with relevant guidelines and regulations for experimental protocols approved under UCSF IRB #19-27586. Seven participants failed to complete their experiments, including three controls, and their data were excluded from the analysis (specifically, three participants withdrew because of nauseous discomfort during their treatment game, and four other participants withdrew because of either loss of interest in the study or unforeseen scheduling problems).

The research is registered at ClinicalTrials.gov, Identifier: NCT04253587, dated 05/02/2020.

#### Neuropsychological testing

All participants enrolled in the study, prior to their recruitment, had already been administered 15 standardized neuropsychological tests that assessed executive and memory functions, and depression. Inclusion criteria for each participant were that all tests scored ≥ -1.5 standard deviations of normative values for their age (Table 1). These evaluations were conducted by UCSF Neuroscape within 6 months of study recruitment and included the following tests: MMSE^47^, geriatric depression (GDS)^48^, working memory and verbal learning (CVLT-II)^49^, processing speed (WAIS-R)^50^, visual-motor sequencing (DKEFS Trail-Making A and B), executive function (DKEFS Stroop interference test), semantic fluency and phonemic fluency (DKEFS)^51^.

#### Overview of experiment procedures

**Figure 2:**
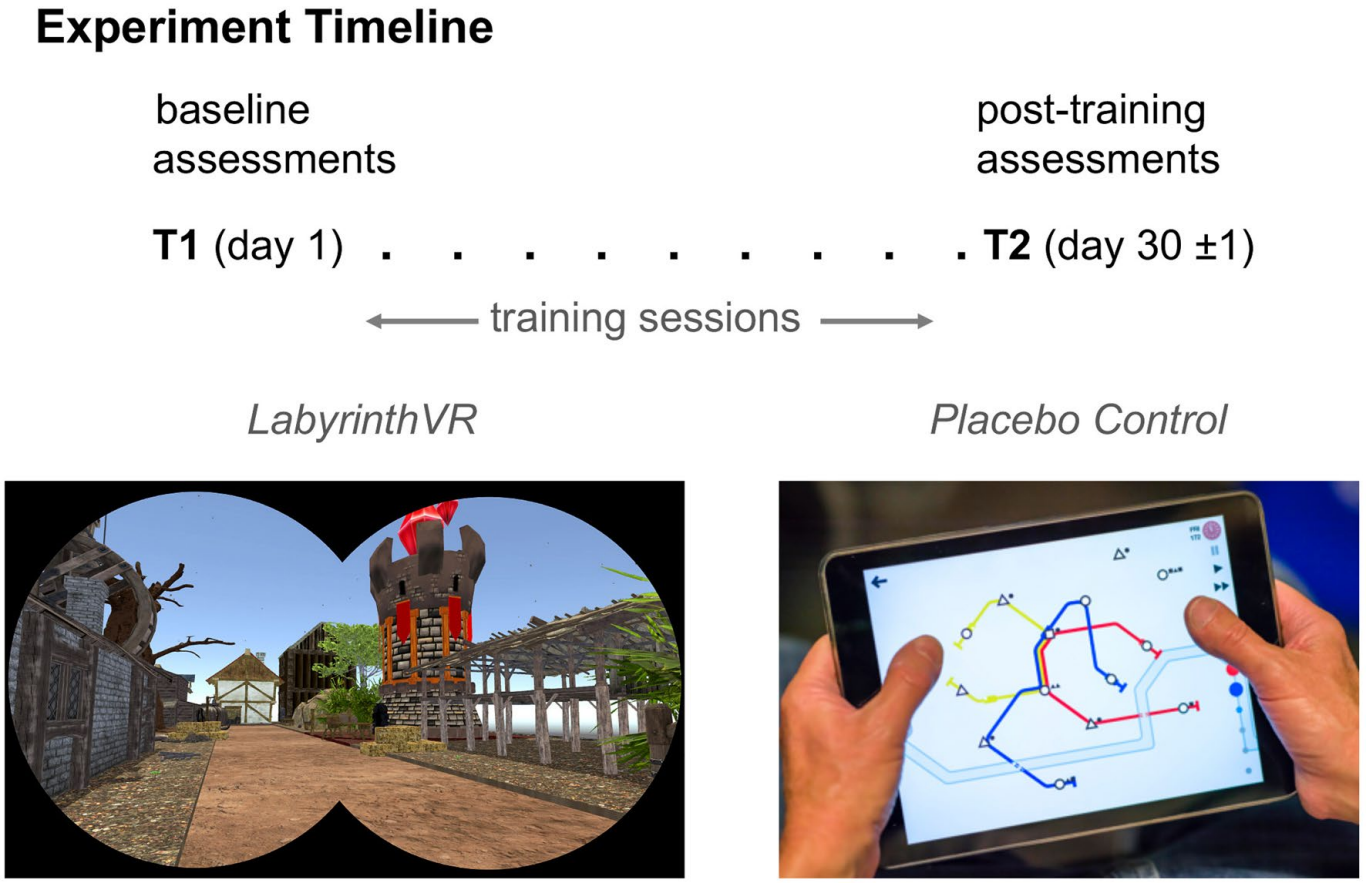
Overview of Procedures in Experiment 2. Participants were randomized to training regimens that included 12 h on task playing either the Labyrinth-VR or the Placebo Control games over 30 days. Baseline and post-training performance on high-fidelity LTM outcomes were assessed promptly before and after each participant’s training regimen.

Recruited participants were randomized to one of two treatment regimens, each of which required a total of approximately 12 h of computer game play in either Labyrinth-VR or the Placebo Control arm (Fig. 2). The first session in a participant’s experiment included, in order, an orientation to their treatment game and schedule, an expectancy questionnaire regarding anticipated results from their treatment regimen, and then a set of tests to assess baseline performance (i.e., pre- treatment, or T1) on LTM outcome measures. Their first session required participants to perform cognitive tasks over approximately 2.5 h, including two brief rest breaks. Participants were kept naïve to the hypothesis for their respective treatment games.

A participant’s treatment schedule began within four calendar days of their T1 assessment. The primary high-fidelity LTM outcome measure was the Mnemonic Discrimination Task (MDT) tested with older and younger adults in Experiment 1. In order to examine a broader gradient in generalization of benefits from Labyrinth-VR treatment to high-fidelity LTM capabilities, additional outcome measures for Recent Autobiographical Recall Task (WALK) and Four Mountains Test (4MT) were also tested. The procedures for these independent measures were sequenced as follows: MDT encoding, then the 30-min WALK encoding, then MDT test, then the WALK test, and finally 4MT.

Once their treatment period was completed, the participant returned within four calendar days for a second round with the same high-fidelity LTM measures in order to assess their post-treatment performance (i.e., T2). Because MDT and WALK test LTM for specific episodic, contextual or temporal order details, we developed two versions of each assessment such that each version required encoding different, although equivalent, sets of stimuli. The order of test versions was counter-balanced within each treatment arm.

#### Expectancy questionnaire

After orientation to their respective treatment games, each participant completed a self-paced, written survey that probed their expectation for gains in cognitive performance as a result from participation in their experiment. The survey posed four questions: Based on your preview of the computer games that you will play during your sessions, do you expect this will affect your performance in terms of: (a) vocabulary, (b) sustaining attention, (c) memory, and (d) navigation? For each domain, select a value to answer as definitely not (1), probably not (2), maybe not (3), maybe help (4), probably help (5), or definitely help (6).

*MDT Outcome Measure Procedure.* The MDT procedure was the same as Experiment 1. An additional stimulus set was selected from the Mnemonic Similarity Task (MST^1^) that was matched in pilot testing to results for the stimuli in Experiment 1 in terms of sensitivity in recognition and discrimination. Our previous research using these MDT procedures and stimuli showed no order effects in discrimination performance between first and second MDT sessions^44^, which plausibly might result from a participant’s familiarity with the recognition test procedure during their second session.

#### WALK outcome measure procedure

The test of recent autobiographical memory (WALK) probes a participant’s recall of individual experiences during a walk in the neighborhood surrounding the UCSF Neuroscape Center, during which the participant hears a scripted narrative from the experimenter leading the walk. In a recall test subsequent to the encoding walk, a verbal questionnaire probes spatial, temporal order and episodic details separately, each of which make autobiographical memories more elaborate^10,11^ .

In the encoding phase, the experimenter leads the participant along a pre-determined, half-mile long route circumscribing the immediate urban neighborhood. Making apparently incidental conversation along the walk, the experimenter actually recites a scripted series of specific comments that relate to five brief stops they make, including personal experience or observations of the experimenter at those landmarks and historical facts for the neighborhood. Each participant hears the same specific comments. Important elements in the script are five stops and three landmarks, which become a source of information for subsequent temporal order questions in the WALK recall test.

The test phase is presented after a 30-min interval, during which the participant completes other cognitive tasks. The test format involves the experimenter presenting a series of five grayscale photographs of the landmarks to the participant, one at a time, and asking seven scripted questions about information learned on the walk at each landmark. The photographs are presented in a pseudo-randomized order, relative to the actual temporal sequence that the landmarks and conversation were learned during the encoding phase. The participants’ spoken responses are transcribed by the experimenter on a score sheet and compared to a list of acceptable correct answers. Calculated from the accuracy of answers to 35 questions, the total recall score possible is 35 points, and partial credit is allowed for correct, but not fully detailed, answers.

Two versions of WALK were developed on the basis of different routes and landmarks. Test questions were matched between versions in terms of the nature of details. Pilot testing showed no difference in the calculated accuracy scores between the two WALK versions on the basis of participants running them independently. The order of WALK versions was counter-balanced within each arm of the experiment.

#### 4MT outcome measure procedure

Spatial memory capability is assessed in the Four Mountains Test (4MT, copyright fourmountains.org.uk) tablet computer game. Although spatial memory is an important capability underlying successful wayfinding, training with Labyrinth-VR involves a broader set of hippocampal-dependent aspects of high-fidelity LTM, such as learning and retrieval of episodic and contextual details. The intent for 4MT as an outcome measure, therefore, is to isolate possible treatment-induced changes in spatial recognition.

4MT results indicate accuracy in recognizing a previously studied, abstract scene of verdant hills in a countryside setting. For each recognition trial, participants are forced to choose one from four alternative views as matching the studied scene, after rotation and changes in the elevation perspective relative to the study target arrangement. The topographical layout of four mountains within a computer-generated landscape is altered in viewpoint, but not colors and textures, relative to the initially presented target images.

There are 15 trials in this short, 4AFC recognition test aimed to assess allocentric-based, spatial memory. Accuracy on the 4MT has been shown to differ between cognitively average older adults and matched MCI patients without dementia^53^.

#### Labyrinth-VR treatment procedure

An HTC VIVE VR system was used with the Unity cross-platform game engine (copyright Unity Technologies) to present our spatial wayfinding game. The Unity developer’s platform affords a myriad of visual assets that can be woven together into a highly detailed, novel VR environ (i.e., an Urban or Village neighborhood). Two neighborhoods were programmed from a large set of Unity visual assets with similar perceptual dimensions. During each participant’s training regimen, each game level (i.e., one, smallest grid, through seven, largest grid) was played in alternating order in the Urban and Village neighborhoods in order to equate a participant’s exposure to the different environs. Game movement in Labyrinth-VR is input from participant ambulation via HTC VIVE position trackers (i.e., “walking in place”). Informed consent for publication of identifying images in an online open-access publication was obtained from our research staff assistant, who demonstrated the game movement procedure in Fig. 1B.

To play Labyrinth-VR, participants walk within the circular perimeter of a platform, which is approximately four feet in diameter and has a waist-high railing. Participants hold a VIVE hand controller, which is visible in VR as a small handheld computer that lists the trial errands by name, elapsed time and also triggers interaction with game elements such as errand completion. Details of the game procedure are explained in the Supplementary Material: Experiment 2.

Route efficiency is the key metric of participant performance in the game. It is measured as the actual distance traversed (including all errands required before exit) versus the optimum route distance calculated from assignment of the trial start point by the game program. Game parameters are designed to provide up to 288 unique trials in a participant’s 15-session treatment regimen, which is scheduled over a 3- to 4-week period according to the participant’s availability. Time on task for each wayfinding session is approximately 45 min, which equates to a treatment dosage of approximately 12 h.

In order to evaluate participants’ wayfinding improvements in the Labyrinth-VR arm over the course of 15 treatment sessions, we developed a simple scale to value each increasing level of trial difficulty with its neighborhood grid size. The scale began at a value of 1 for Level 1 in Grid 1 and reached maximum at 42 for Level 5 of Grid 7. Each participant’s game achievement in Labyrinth-VR was measured as the value for their highest level at or above the route efficiency threshold.

#### Placebo controls treatment procedure

The Placebo Control condition uses four commercially available computer games (i.e., Syberia by Microids, Burly Men at Sea by Brain & Brain, Reigns by Devolver Digital, and Mini Metro by Dinosaur Polo Club), which each involved their own narratives to create game goals, but do not require remembering detailed information or route navigation in order to achieve success. Particular consideration in the final selection of control games was given for the visual complexity of the stimuli in each game environment, so as to best match appearances with Labyrinth. In Syberia and Burly Men at Sea, for example, the details of game scenes depicted color, texture, varying lighting conditions and often conveyed large-scale spaces. Mini Metro, as a different example, demanded construction and balancing of resources across increasing complexities on a topological map. In sum, we selected control games that were functionally similar to the Labyrinth wayfinding game in terms of visual experiences. The control games relied on some form of interaction with minute-by-minute game goals, but substantially different from Labyrinth in terms of demands to form memories from which participants might build allocentric neighborhood maps. Details of the control arm procedure are explained in the Supplementary Material: Experiment 2, including representative screen grabs.

The placebo games had been selected in a preliminary study with a separate group of volunteers. We used an empirical assessment of volunteers’ expectations of treatment-related gains in cognition to find a combination of iPad games that would act as an appropriate placebo control for our Labyrinth intervention. Expectancy matching involved showing volunteers a 10-min orientation of either Labyrinth-VR or a selection of placebo games before they completed a written survey. The survey asked them to rate their expectation that treatment with the orientation game would lead to performance improvements in each of four domains (see Expectancy Questionnaire above). Based on the survey responses from the first 10 volunteers, the selection of iPad games was narrowed from six to four. A second group of 10 volunteers repeated the procedure, and their expectancy results did not differ whether they had been oriented with Labyrinth-VR (mean rating = 3.0 ± 1.1) or the four placebo games (mean rating = 2.8 ± 1.7).

Data were analyzed and statistics calculated using IBM SPSS Statistics release 20.0.

### Results

Participants were assigned at random to treatment arms using either Labyrinth-VR or the placebo control games. Comparison of the participant data collected at neuropsychological assessments found no differences in mean baseline performance between the Labyrinth-VR and placebo control arms in the six cognitive tests most relevant for LTM and cognitive control (Table 1).

Individualization of each participant’s treatment schedule was permitted to accommodate his/her availability to commute to the Neuroscape laboratory, providing that all treatment sessions and outcomes assessments were completed within 40 calendar days. For Labyrinth-VR, mean treatment duration before T2 assessment was 29 ± 5 calendar days. For Controls, mean treatment duration before T2 assessment was 30 ± 6 calendar days.

Expectancy ratings were collected at the end of each participant’s orientation session, which provided their predictions of whether the assigned treatment regimens would help cognitive performance, based on responses along a six-point Likert scale. The ratings showed that participants’ expectancy for treatment-related improvements did not differ between the Labyrinth-VR and Controls game arms for Vocabulary (Labyrinth-VR 3.5 ± 1.9; Controls 2.7 ± 1.3; *t*_19_ = 1.49, *p* = 0.14), Sustaining Attention (Labyrinth-VR 4.9 ± 1.0; Controls 4.7 ± 1.6; *t*_19_ = 0.54, *p* = 0.58), Memory (Labyrinth-VR 5.0 ± 0.8; Controls 4.6 ± 1.4; *t*_19_ = 1.14, *p* = 0.25), or Navigation (Labyrinth-VR 5.0 ± 0.8; Controls 5.0 ± 1.6; *t*_19_ = 1.32, *p* = 0.20). Participants’ expectations for treatment-related gains were matched between the Labyrinth-VR and Controls arms after consideration of their orientation session experiences and before their interventions began, thus establishing the control games as appropriate placebos for the Labyrinth-VR treatment.

Mean overall achievement score on the Labyrinth-VR game itself was 31.81 ± 5.51, and a comparison between Urban and Village neighborhoods, as repeated measures, showed no difference in achievement (Urban = 30.91 ± 5.70; Village = 31.80 ± 6.15; *t*_20_ = 1.43, *p* = 0.16). Participants’ overall achievement scores from training were used in the further analyses of individual differences.

Additionally, considering a broad pattern in the LTM literature that males often perform spatial navigation and wayfinding tasks more efficiently than females^54–56^, we compared the participants’ games achievement scores by gender. This test found no difference in achievement on Labyrinth-VR as based on the participant’s gender (females = 29.57 ± 6.05; males = 32.93 ± 5.08; *t*_10_ = 1.26, *p* = 0.23).

#### MDT outcome

The primary analysis examined results for the high-fidelity LTM test, which all participants completed at their T1 and T2 assessments, between Labyrinth-VR and Controls. In MDT, the mnemonic discrimination task that used pictures of common objects, the key outcome for each participant was their lure discrimination index (LDI). The index corrects a participant’s raw correct rejection rate for lures (i.e., true negatives) with their false alarm rate to novel images (i.e., false positives), such that LDI = proportion LureCR—proportion NovelFA.

For the analysis of LDI, mixed design ANOVA (repeated factors for time T1|T2 X between factors for condition Labyrinth-VR | Controls) revealed a main effect of condition (*F*1,20 = 5.493, *p* = 0.030, Cohen’s *d* = 0.71) and an interaction of condition and time (*F*1,20 = 6.941, *p* = 0.016, Cohen’s *d* = 0.79). Pairwise comparisons showed LDI increased for Labyrinth-VR (T1 = 0.57 ± 0.03, T2 = 0.66 ± 0.03, *p* = 0.001), whereas Controls showed no change (T1 = 0.60 ± 0.03, T2 = 0.59 ± 0.03, p = 0.84). There was no main effect of time (*F*1,20 = 0.444).

**Figure 3:**
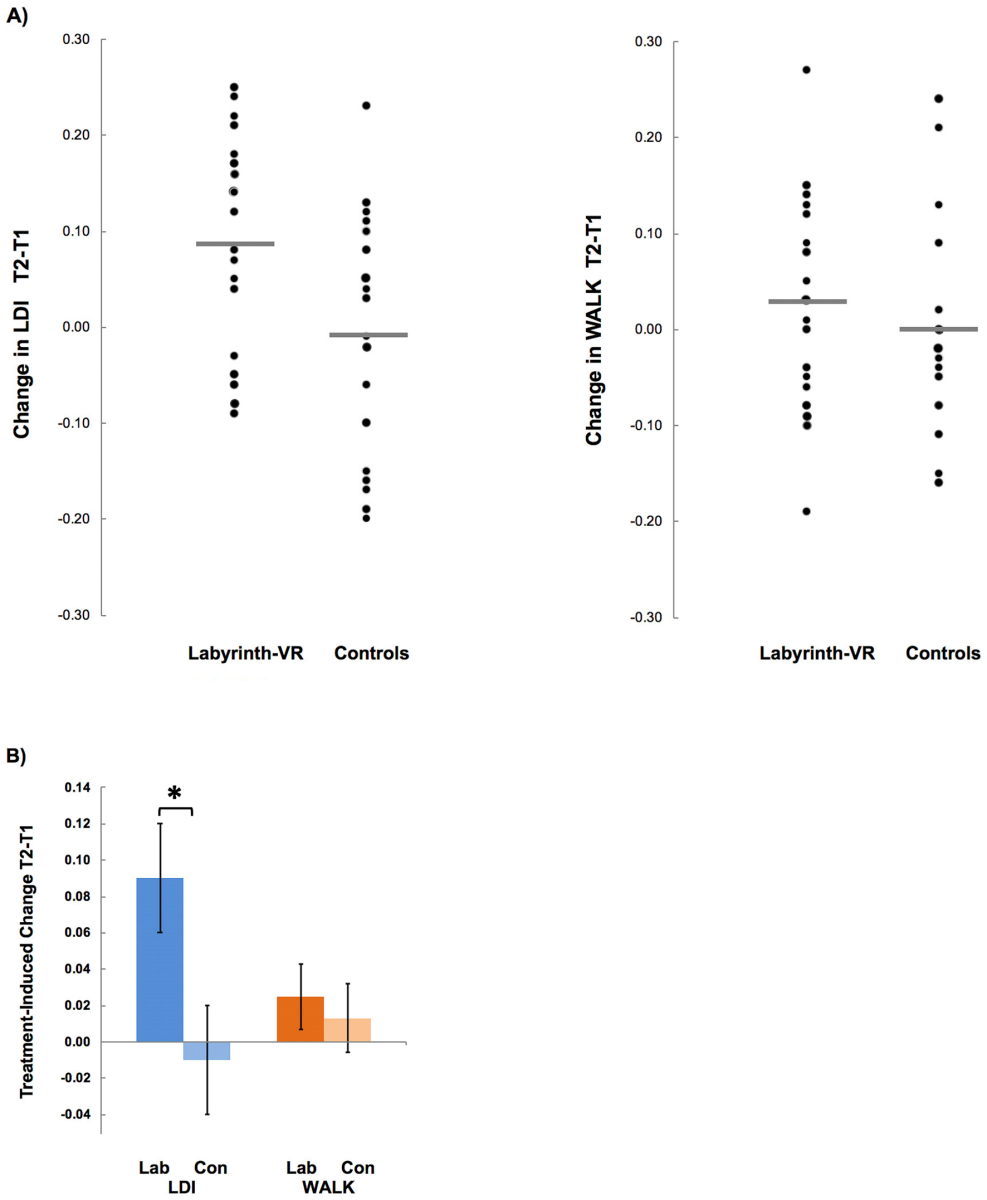
Results for LTM Outcome Measures. (A) The distributions of participants’ individual change scores (i.e., T2-T1) are presented for the Labyrinth-VR and Control arms from the results for Lure Discrimination Index (LDI) and WALK. Mean performance in each column is shown by a horizontal bar. (B) Comparisons of treatment-induced changes between arms showed a robust gain in LDI for Labyrinth-VR (Lab), relative to Control (Con), and a smaller numerical gain in WALK for Lab, relative to Con. * indicates a difference between arms, p < 0.05.

Subtraction of a participant’s T1 assessment score from their T2 assessment score showed changes in LDI resulting from their treatment, which indicated effects of their intervention on this untrained outcome measure (Fig. 3A). These results showed the robust effect that the Labyrinth-VR regimen induced improvement of an important, untrained capability for high-fidelity LTM retrieval (Fig. 3B), relative to placebo controls.

#### Additional LTM outcomes

Performance between treatment arms in MDT, WALK and 4MT were each compared separately, as these three tests measured different qualities of high-fidelity LTM retrieval with entirely independent procedures and stimuli, and we did not predict that there would be reliable inter-correlations between these measures.

For WALK, the recent autobiographical memory task, the key outcome for each participant was the proportion of correct answers recalled for 35 questions cued only by a set of generic, wide-angled, grayscale photographs depicting scenes from the WALK routes. Mixed design ANOVA did not show an effect of condition (*F*1,20 = 1.616, *p* = 0.22), of time (*F*1,20 = 0.613, *p* = 0.44), or an interaction of condition and time. Similarly, for 4MT, mixed design ANOVA did not show an effect of condition (*F*1,20 = 1.85, *p* = 0.19), of time (*F*1,20 = 0.004, *p* = 0.95), or an interaction of condition and time.

#### Individual differences

**Figure 4:**
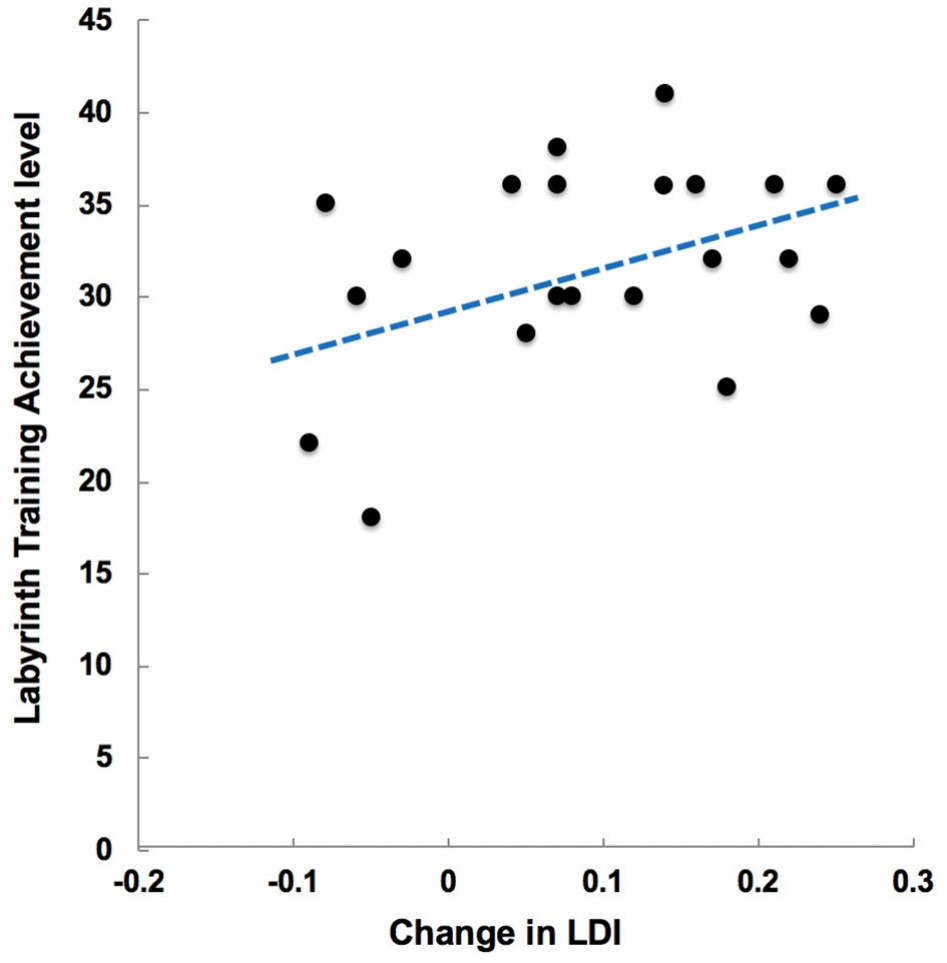
Individual Differences. Relative to their game achievement level, individual change scores in LDI (i.e., T2-T1) are presented for participants in the Labyrinth-VR treatment arm. A trend was evident between higher Labyrinth achievement and treatment-induced gain in LDI (*p* = 0.10).

In order to assess if a participant’s advancement in the Labyrinth-VR wayfinding game was predictive of the magnitude of their improvement in high-fidelity LTM retrieval, a follow-up analysis of data for Labyrinth-VR participants compared individual differences in their respective game achievement with their gains in the LTM outcomes. Results showed a trend toward a significant correlation between greater Labyrinth-VR achievement with greater improvement in the MDT outcome, such that participants who progressed to higher levels of wayfinding challenge also realized greater gains in LDI (*r*20 = 0.37, *p* = 0.10, Fig. 4). Individual differences in the participants’ Labyrinth-VR achievement did not predict WALK outcome (i.e., recent autobiographical recall, T2-T1) (*r*20 = -0.20, *p* = 0.380). Investigating these data further showed that the Labyrinth-VR arm participants achieving at least game level 38, out of 42 possible, realized treatment-induced gains in both LDI and WALK.

### Analysis of results across Experiments 1 and 2

#### Comparisons of LDI across younger, older and Labyrinth-VR participants

The results from Experiment 1 showed that LDI is a sensitive measure for age-related changes in mnemonic discrimination (Table 2). In order to further examine the effects of our VR treatment, we compared mean LDI scores from the Labyrinth-VR participants, who were all older adults (see Experiment 2), with the mean LDI scores for groups of younger and older adults that had completed the same MDT task, using the same stimuli and procedure (see Experiment 1). Notably, mean performance was not different between the Labyrinth-VR participants at baseline (i.e., T1 LDI = 0.57 ± 0.03) and the group of older adults from Experiment 1 (LDI = 0.52 ± 0.04) (*t*_44_ = 1.17, *p* = 0.25, independent variables test with unequal variances).

**Figure 5:**
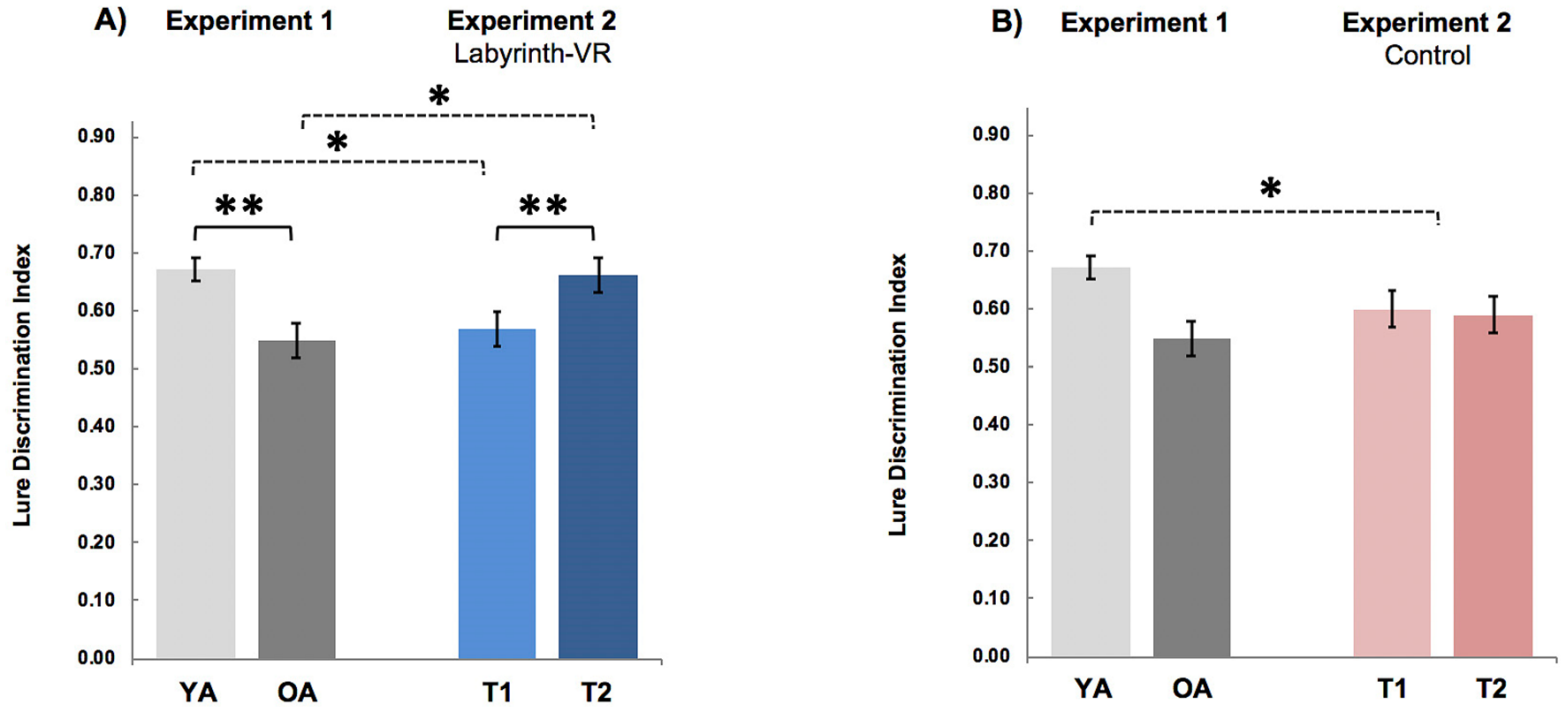
Comparisons of LDI for Younger, Older and Labyrinth-VR Participants. (A) Mean LDI scores for participants in the Labyrinth-VR arm in Experiment 2 (i.e., at baseline, T1, and post-training assessments, T2) are compared to the mean LDI scores for groups of younger and older adults that completed the same Mnemonic Discrimination Task in Experiment 1. Labyrinth-VR participants at T1 showed a diminished level of LDI typical of older adults (OA), and then they improved this important high-fidelity LTM capability at T2 up to a level typical for younger adults (YA). (B) Mean LDI scores for participants in the Control arm in Experiment 2, at both T1 and T2, were below the mean level of YA in Experiment 1. ** indicates a difference between means, p < 0.001, and * indicates a difference between means, p < 0.05.

Based on the results from Experiment 1, we expected baseline performance (i.e., T1) for the Labyrinth-VR participants would show diminished high-fidelity LTM capability relative to younger adults. We also hypothesized that post-treatment performance of the Labyrinth-VR participants would improve to LDI levels similar, on average, to younger adults. Results from our post-hoc comparisons found that the Labyrinth-VR participants at baseline scored below the younger adults mean LDI = 0.67 ± 0.02 (*t*_44_ = 2.46, *p* = 0.02, Cohen’s *d* = 0.73; all comparisons between experiment groups were independent variables tests with unequal variances). At post-treatment (i.e., T2), LDI performance for the Labyrinth-VR participants improved to the level of the younger adults (*t*_44_ = 0.13, *p* = 0.90) (Fig. 5A). For the Control participants, however, LDI performance at baseline was below the younger adults mean (*t*_44_ = 2.20, *p* = 0.03, Cohen’s *d* = 0.66) and remained unchanged at post-treatment assessment (*t*_44_ = 2.11, *p* = 0.04) (Fig. 5B).

In summary, comparisons for the effects of the Labyrinth-VR intervention on mnemonic discrimination found that older participants, who, on average, had shown an age-typical level of diminished LDI at baseline, then improved this important high-fidelity LTM capability after treatment up to a level typical for younger adults. This pattern in improvement suggests that the Labyrinth-VR intervention can restore high-fidelity LTM capability for older adults to the typical level of younger adults.

## Discussion

After treatment for 12 h in the Labyrinth spatial wayfinding VR game, older adults with average cognitive performance for their ages demonstrated reliable gains in an untrained measure of high-fidelity LTM retrieval, relative to matched older adults who played placebo control games. This report provides the first evidence from a randomized control study supporting our concept for an intervention that improves high-fidelity LTM ability in older adults.

Importantly, we found that among three groups of healthy older adults showing equivalent mnemonic discrimination ability at their baseline tests, only the post-treatment group that engaged in Labyrinth-VR showed LDI performance equivalent to the level of younger adults (i.e., comparing results as shown in Fig. 5). The results suggest that a cognitive intervention can remediate age-related deficits in high-fidelity LTM experienced by older adults. We developed Labyrinth as a VR computer game such that it allowed for rigorous control of the presentation of rich, detailed and entirely novel learning experiences for all participants. We hypothesized such a form of environmental enrichment would provide a therapeutic intervention to treat LTM decline in aging.

Neither cognitive interventions, nor pharmaceutical treatments, are available today to restore high-fidelity LTM capabilities, which decline in normal aging and are substantially impaired at the outset of Alzheimer’s Disease^4^. Decline in high-fidelity LTM capabilities for older adults is directly linked with diminished hippocampal function, whether or not directly attributable to a neurodegenerative condition^57^. The Labyrinth-VR intervention is aimed to restore high-fidelity LTM capabilities via adaptive training in a task that engages with hippocampal-dependent LTM functions.

### Immersive and engaging training procedure

The novelty of our approach to use VR and game movement by active ambulation was aimed at creating a very engaging, immersive and adaptive wayfinding challenge. The 3D functionality of VR provided for synchronization of game movement with participants’ head movements and associated walking motion. In sum, VR imbued greater ecological validity in the wayfinding challenges, which we believe promoted deep engagement in the game. The particular focus was to sustain participants’ enthusiasm for learning and performing assigned errands over 15 sessions, so as to stimulate the greatest beneficial effects from their environmental enrichment. Framed from findings about participant engagement detailed in the psychometric literature^58^, the treatment regimen incorporated an adaptive scale based on efficient wayfinding success with feedback (i.e., route efficiency) to raise task difficulty up to 42 possible levels^29^.

Movement through the virtual environment was co-registered from a participant’s ankle motions while walking in place, an approach that applied ecologically valid motoric input during the VR training procedure. The game software incorporated hundreds of visual and auditory stimuli as the means to create entirely novel neighborhoods for participants to learn. Night-time and fog-shrouded game trials, when color, texture and perspective for route landmarks were substantially degraded, were the most difficult challenges for the accuracy of participants’ allocentric navigation in the new neighborhoods. All of these elements in the Labyrinth-VR game were expected to contribute to participants’ engagement and interaction with the wayfinding task, so as to optimize their opportunity for enrichment by increasing demands on hippocampal-dependent learning. In the video gaming literature, scientific examination of a similar construct for interactivity has tested the notion that HMD VR makes multidimensional demands for a player’s engagement with the game^59^. Future work comparing Labyrinth game versions without HMD VR, or interactive ambulatory game movements, will be needed in order to better understand which demands are most effective in driving and sustaining a participant’s engagement with the training intervention and its effect on cognitive mechanisms.

Our approach was based on findings from both human and animal research that show exploration of novel and complex environs (i.e., environmental enrichment) results in improved capability for hippocampal-dependent LTM^23,25,28^. Direct evidence of treatment-induced gains in hippocampal function necessary for high-fidelity LTM is available from studies with animal^21,22^, which show increased neurogenesis, synaptogenesis and up-regulation of neurotrophic factors. Recent results from human histological data show that hippocampal neurogenesis persists in normal aging^60^, specifically regions of the dentate gyrus. The convergence of findings about adult-borne dentate granule cells from human and animal models provides the fundamental mechanism by which treatment-induced up-regulation of hippocampal function could bring gains in high-fidelity LTM capabilities.

### Qualitative measures of high-fidelity LTM

Several measures of high-fidelity LTM have been shown to depend on hippocampal functions^6,20,22,43^, and those findings suggest that episodic, spatial, contextual and temporal order memories are processed in distinct functional units of the hippocampus^42^. We assessed treatment-induced changes in high-fidelity LTM using a primary outcome measure for mnemonic discrimination, which was collected in Experiments 1 and 2. Additional LTM measures were assessed in Experiment 2 (i.e., recent autobiographical memory and spatial recognition) in order to examine the potential for a broader range of generalization from treatment-induced gains to other high-fidelity LTM capabilities. In our cohort, Labyrinth-VR treatment resulted in reliable gains only in mnemonic discrimination. Drawing upon other results associated with functional neuroimaging, we consider relevant points for this pattern in the behavioral outcomes here as related to possible target engagements in the high-fidelity LTM system.

The MDT outcome measure tests a participant’s ability to discriminate highly similar lures from studied targets, which provides a behavioral readout of the efficacy of an underlying pattern separation process^13,61^. Behavioral pattern separation is associated with hippocampal function^14^, in particular the dentate gyrus/CA3 regions, and this capability has been shown to decline in normal aging. fMRI results show aging-related disruption in dentate gyrus/CA3 function is associated with diminished LDI^15^, relative to younger adults. Changes in LDI, therefore, may serve as a proxy for dentate gyrus/CA3 function, including an indication of change in dentate neurogenesis^62^. Taking these findings into account, a parsimonious interpretation of our results here would be that gains in LDI induced by Labyrinth-VR likely arose from sustained engagement with dentate gyrus/CA3 function. This inference holds well in terms of both longitudinal comparisons in Experiment 2 and cross-sectional comparisons between Experiments 1 and 2.

The WALK outcome measure tests for breadth and richness in recent autobiographical memory, and, consequently, it assesses capability for high-fidelity LTM that draws on self-referential encoding. The Self-Referential Effect (SRE)^63^ in LTM is a well-developed model to distinguish how objective recollection can integrate episodic, source and contextual information around personal experiences. The richness of SRE may be diminished in older, relative to younger adults^64^, and such decline has been associated with changes in medial prefrontal and precuneus functions^65,66^. Other reports, however, have concluded that self-referential memory processes remain intact in healthy aging^67^, and then even in relationship to declines an older adult may suffer in other measures of high-fidelity LTM^68^ . This possibility is attributed to the interpretation that SRE may depend as much on associative memory processing in cortex as on hippocampal function^69^.

WALK specifically engages participants in self-referential encoding, and then tests for more elaborate, temporally detailed retrieval in high-fidelity LTM than lure discrimination (i.e., MDT). Notably, retrieval processes associated with source memory, on the one hand, and behavioral pattern separation associated with lure discrimination, on the other hand, have recently been shown to engage distinct hippocampal and neocortical regions in younger adults^42^. In the present study, results showed a weak group effect of treatment on WALK in the Trackers arm, as only 12 of 21 participants improved their WALK scores from baseline to post-treatment assessments. Although these findings may indicate that Labyrinth-VR did not substantially interact with SRE, further study is necessary to validate whether greater dosage of Labyrinth-VR treatment could benefit SRE or other measures of high-fidelity LTM that we did not assess in this study.

The 4MT outcome measure was developed to test spatial recognition after manipulating the topographical orientations of a studied spatial scene^70^. Although playing Labyrinth-VR challenges allocentric navigation, rather than spatial recognition, we hypothesized that treatment in the former may benefit the latter. The null treatment effects with 4MT suggest that the processes involved when forming a mental map for a neighborhood in LTM do not necessarily enhance the short-term memory processes involved in maintaining topographical orientation.

### Limitations

A few limitations in our procedures and results should be weighed with caution when interpreting the novel findings presented. In particular, we discuss points relevant to our control group and dosage.

Interventional results must always be interpreted with caution regarding their therapeutic effects relative to placebo, which can theoretically be controlled across a gamut of mechanistic and expectancy elements^71,72^. Here, the placebo control was a set of commercially-available, narrative computer games, all of which included novel and engaging visual stimuli. Although these games presented amusing story lines and were visually enriched, their game goals placed minor demands on LTM, if any. We selected the most naturalistic treatment environment for the comfort of participants in order to encourage their engagement in their treatment regimen (i.e., iPads played primarily at home on a regular schedule they set).

Critically, after game orientations in each arm, we surveyed participants’ expectancy for cognitive effects that they anticipated from their treatment regimens. The survey results show that, at baseline, expectancy for treatment-induced improvements in memory, sustaining attention, navigation and vocabulary were all matched between arms. We are confident that the results from Experiment 2 show a significant improvement beyond that attained by an active, expectancy-matched control group and, therefore, rule out practice effects and placebo effects as the source of benefit. Nonetheless, future research is needed to explore the specific role of the VR environment in yielding the positive outcomes, as opposed to the same navigation challenges being presented in a 2D environment, and also without ambulation.

Dosage of treatment was established after six treatment experiments with a pilot version of Labyrinth-VR and consideration of relevant data published on cognitive interventions with healthy older adults. Preliminary piloting suggested that 10 h on task was effective for most participants. In order to be confident in achieving saturation across a full experiment sample, however, we opted for dosage that would meet or slightly exceed 12 h on task over 15 one-hour treatment sessions. Similarly, another computer game intervention with healthy older adults applied the same dosage of 12 h treatment on task over approximately four weeks and found effective saturation in a sample size of 46 healthy older adults^73^.

Mean game achievement after 12 h on task was 31.81 ± 5.51, or approximately level 32 out of 42 possible. Yet, our results show that five of 21 Labyrinth-VR participants did not realize treatment-induced gains in LDI and had mean game achievement of only level 24. The nine of 21 participants who did not realize treatment-induced gains in WALK had mean game achievement of level 33. Further comparisons found that of the seven participants surpassing game level 35, five showed training-induced gains in both LDI and WALK. Both participants surpassing game level 37 showed training-induced gains in both LDI and WALK. Comparisons of individual differences in game achievement and LTM outcome scores indicate that dosage should be increased in order to achieve better treatment saturation (Fig. 4). A flexible and personalized approach aimed to achievement of at least game level 38 may be the ideal implementation of Labyrinth-VR as a treatment.

Future studies with Labyrinth-VR will be needed to better understand the subtleties of active ingredients that induced the treatment effects reported here. The training procedure involved both VR and mild exercise, and more work could control between these factors in order to determine the relative benefits coming from visual immersion by the head-mounted display, or game movement via ambulation.

In summary, the findings here show restoration of high-fidelity LTM capability for healthy older adults with average cognitive performance, such that their performance improved to a level similar to younger adults. Our report is a novel example of a cognitive intervention that promises help for LTM decline associated with aging, and it represents a beachhead from which to expand research with both healthy and cognitively impaired older adults.

## Supplementary Information

Supplementary Information.
