# Supplementary material for: Virtual reality video game improves high-fidelity memory in older adults
Source: Sci Rep. 2021 Jan 28;11:2552. doi: 10.1038/s41598-021-82109-3 (PMC7844043; doi:10.1038/s41598-021-82109-3)
Supplement: Supplementary file 1 — Supplementary Information. [file 41598_2021_82109_MOESM1_ESM.docx]

**Supplementary Information for:**

**Virtual Reality Video Game Improves High-Fidelity Memory in Older Adults**

**Authors**

Peter E. Wais^1^*, Melissa Arioli^1^, Roger Anguera-Singla^1^ & Adam Gazzaley^1,2^

**Affiliations**

^1^ Department of Neurology, Neuroscape and Weill Institute for Neurosciences,

University of California, San Francisco

^2^ Departments of Physiology and Psychiatry,

University of California, San Francisco

***Communicating author:** Peter Wais [peter.wais@ucsf.edu](mailto:adam.gazzaley@ucsf.edu)

UCSF – MC0444

675 Nelson Rising Lane

San Francisco, CA 94158

Tel: 1-415-502-7322

**Supplementary Material**

**Experiment 1.**

The mnemonic discrimination task (MDT) applied here was adapted from our previous published studies that used this test in association with collection of neuroimaging data

with younger adults ^6,27^, and it has been adapted in other published reports examining effects with older adults ^1,8,43^.

MDT is a behavioral pattern separation task used to assess a key capability of high-fidelity LTM retrieval (**Figure S1**). Discrimination between a target and its paired lure requires memory of the details unique to the studied target, which leads to recognition of a target as “old.” Correct rejection of a similar lure as “new” indicates a memory judgment based on discrimination of the differences from the studied target, which likely involves specific details relevant to the target. In contrast, a false alarm to a similar lure as “old” indicates mistaken recognition, which is likely based on a simplified match in memory with the gist of the details of the paired target.


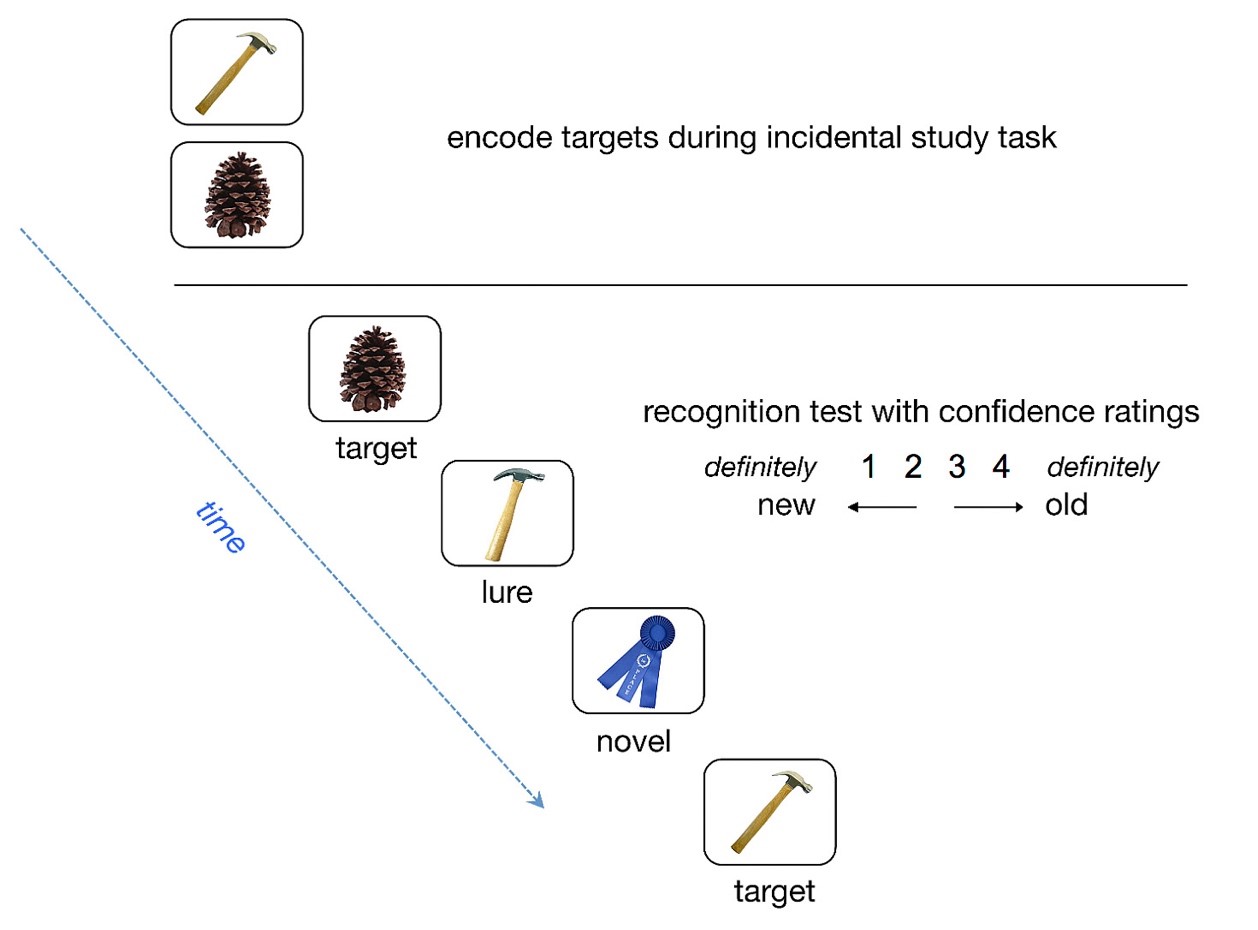


**Figure 1S.** The schematic illustration presents the experimental paradigm for the mnemonic discrimination task (MDT).

**Experiment 2.**

*Labyrinth-VR Treatment Procedure.* The protocol for each Labyrinth-VR treatment session provides for participant safety and comfort with consideration for the level of exertion required for ambulation in the game. At the beginning of each session, the participant warms up with a 180s video that presents stretching exercises on a 65” monitor in the motion-capture experiment room. After warm-up, the 60-minute session begins, which typically provides 45 minutes on task. To play Labyrinth-VR, participants walk within the circular perimeter of a platform, which is approximately 6 feet in diameter and has a waist-high railing. Participants hold a VIVE hand controller, which is visible in VR as a small handheld computer that lists the trial errands by name, elapsed time and also triggers interaction with game elements such as errand completion.

The game storyline begins with arrival in an unfamiliar neighborhood at a randomized starting point, from which the participant learns to navigate through virtual Urban or Village environments during a 10-minute exploration (i.e., neighborhood encoding). Before encoding, the participant is given simple instructions to locate and remember the errand locations and game exit point for that neighborhood, while forming “a mental map of the area and its landmarks.” Errands are highlighted with visual and auditory alerts during encoding, but not on wayfinding trials. Streets and pathways are not named. An additional learning period can be added at the participant’s option. Streets and pathways are not named. Test trials then follow encoding immediately, when the participant is instructed to locate specific errand(s) and proceed to the exit point, all the while using the most efficient route (i.e., fewest number of steps). Each trial requires completing one, two or three errands dispersed in the neighborhood before making way to the exit location, and gamification of successful errand completions and exiting the trial provides rewarding visual feedback.

Test trials then follow encoding immediately, when the participant is instructed to locate specific errand(s) and proceed to the exit point, all the while using the most efficient route (i.e., fewest number of steps). Each trial requires completing one, two or three errands dispersed in the neighborhood before making way to the exit location, and gamification of successful errand completions and exiting the trial provides rewarding visual feedback. Route efficiency is the key metric of participant performance in the game. It is measured as the actual distance traversed (including all errands required before exit) versus the optimum route distance calculated from assignment of the trial start point by the game program. When a user completes all assigned errands, reaches the game exit within 7 minutes and has not exceeded 1.25x the optimum route (i.e., an 80% route efficiency threshold), then game feedback will indicate a successful trial and trigger advancement to the next level. Failure to complete the errands within the time limit, or to take an efficient route, will result in game feedback to try again, and the level will restart at a new, pseudo-random spawning point.

Movement is measured in terms of traversing “tiles,” each of which simulates approximately 10m X 10m of street surface including adjoining sidewalks. At the conclusion of a trial, a feedback screen reports route efficiency and trial outcome, which are the determining values for triggering adaptive steps in the level of challenge in the wayfinding game. When a user completes all assigned errands, reaches the game exit within 7 minutes and has not exceeded 1.25x the optimum route (i.e., an 80% route efficiency threshold), then game feedback will indicate a successful trial and trigger advancement to the next level. Failure to complete the errands within the time limit, or to take an efficient route, will result in game feedback to try again, and the level will restart at a new, pseudo-random spawning point.

After succeeding at all errand levels in a neighborhood, a visual summary provides user feedback that includes a recap of route efficiencies for all trials run and traces of the actual routing taken on those trials (projected onto the overhead perspective map from the demonstration view, **Fig. 1A**). The user then advances to a new, larger neighborhood with new errands. The grid sizes are 1.00X, 1.33X, 1.70X, 2.10X, 2.60X, 3.60X and 4.98X. Depending on grid size and level, optimal trial routes range from a minimum of 19 to a maximum of 150 tiles. Game parameters are designed to provide up to 288 unique trials that can be drawn into a participant’s 15-session treatment regimen, which are scheduled over a three- to four-week period according to the participant’s availability.

Labyrinth-VR includes trials in two environs (i.e., Urban and Village), each of which draws seven neighborhood grids of increasing size and complexity in order to build an extended, adaptive challenge for each environ. Two environs were developed in order to increase novelty in the wayfinding task, and therefore, engagement in learning. Urban and Village neighborhoods are alternated sequentially during the treatment regimen. For example, in the Urban Environ, a mixed cityscape of taller, contemporary buildings and two-story, older structures appears on a rectilinear grid of streets. Color and texture are greatly varied across structures in each neighborhood. The most challenging levels in each neighborhood present lighting of a mildly moonlit night, and then dense fog that nearly eliminates structures’ colors and textures as landmark cues.

After succeeding at all errand levels in a neighborhood, a visual summary provides user feedback that includes a recap of route efficiencies for all trials run and traces of the actual routing taken on those trials (projected onto the overhead perspective map from the demonstration view, Fig. 1A). The user then advances to new neighborhoods with new errands, and grid sizes progress as 1.00X, 1.33X, 1.70X,

2.10X, 2.60X, 3.60X and 4.98X.

*Placebo Controls Treatment Procedure.* The placebo games were played on a 9.7” Apple iPad in a dosage-matched treatment regimen, which participants in the control arm completed mostly at home. The selection of four games was included so that individual control participants could enjoy a range of choices about which games to play for repeated hours, but they were instructed to complete two 1-hour sessions in each game before choosing which games to play for the remainder of their regimen. Allowing participants to select the number of hours played on each game, or even avoiding playing a disliked game after trying it out, was aimed at matching engagement during treatment regimens between the Labyrinth-VR and placebo control groups.

Participants randomized to placebo controls treatment were instructed to play their selected games for 12 one-hour sessions, and they were later debriefed about their game preferences and progress. Time on task for each of the control sessions was approximately 60 minutes, which equates to a treatment dosage of approximately 12 hours.


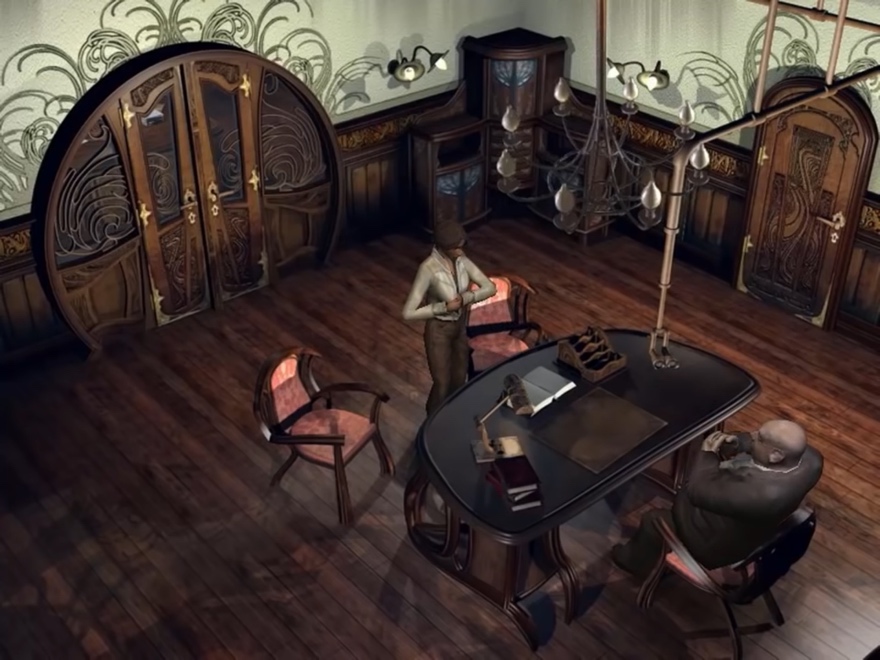

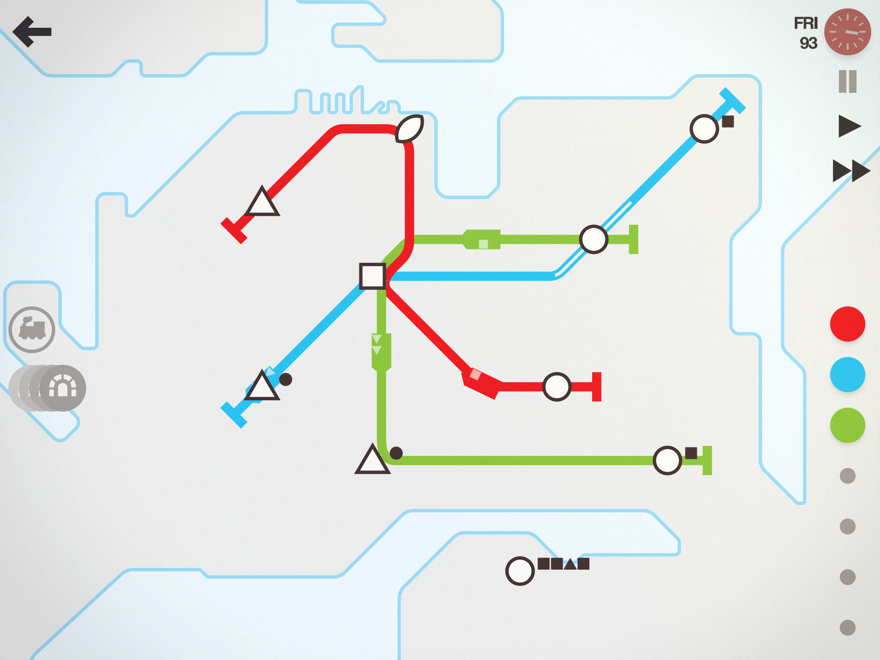


**Figure 2S.** Representative screen shots from placebo control games tablet user interface are presented from Syberia by Microids and Mini Metro by Dinosaur Polo Club, respectively. The Syberia written narrative presents a mystery for the participant to solve, while Mini Metro presents dynamic changes in passenger loads for the participant to balance.
